# Prolonged shedding of severe acute respiratory coronavirus virus 2 (SARS-CoV-2) RNA among patients with coronavirus disease 2019 (COVID-19)

**DOI:** 10.1017/ice.2020.307

**Published:** 2020-06-24

**Authors:** Jessica P. Ridgway, Nirav S. Shah, Ari A. Robicsek

**Affiliations:** 1 Department of Medicine, University of Chicago, Chicago, Illinois; 2 NorthShore University HealthSystem, Evanston, Ilinois; 3 Providence St. Joseph Health, Renton, Washington

Early reports from China indicate that severe acute respiratory coronavirus virus 2 (SARS-CoV-2) RNA may persist in the respiratory tracts of patients with coronavirus disease 2019 (COVID-19) for several weeks after symptom onset.^1–3^ However, the duration of SARS-CoV-2 RNA shedding has not been systematically studied in a large cohort of patients.

## Methods

To estimate the duration of SARS-CoV-2 RNA shedding, we conducted a multisite study among patients who had nasopharyngeal specimens tested for SARS-CoV-2 RNA via real-time polymerase chain reaction (PCR) assay at Providence St Joseph Health (a 51-hospital healthcare organization based in Renton, Washington), University of Chicago Medicine in Chicago, Illinois, and NorthShore University HealthSystem (a 5-hospital healthcare system based in Evanston, Illinois). All patients with a positive SARS-CoV-2 PCR test between January 22, 2020, and April 23, 2020 who had at least 1 subsequent SARS-CoV-2 PCR test were included in the study. SARS-CoV-2 PCR tests were ordered at the discretion of medical providers at each institution. We calculated the percentage of patients with a persistent positive SARS-CoV-2 PCR test result up to 25 days after the first positive test. This study was approved by the institutional review board of each institution.

## Results

During the study period, 76,040 SARS-CoV-2 PCR tests were performed among 70,406 unique patients. The mean age of all patients tested was 48.3 years. Of these patients, 10,584 (15%) tested positive for SARS-CoV-2. Of these 10,584 patients, 555 (5%) with an initial positive test for SARS-CoV-2 RNA underwent at least 1 subsequent SARS-CoV-2 PCR test within 25 days of the first test. The mean age of patients who tested positive and had a subsequent test was 61.7 years. Among 156 patients with a subsequent test 1–5 days after their initial positive test, 138 (88%) continued to have a positive test (Table 1). Among 105 patients with a subsequent test 21–25 days after their initial positive test, 59 (56%) continued to have a positive test.

**Table 1.** Duration of SARS-CoV-2 RNA Detection

No. of Days After 1^st^ Positive SARS-CoV-2 PCR Test	Subsequent Positive SARS-CoV-2 PCR Tests, No./Total (%)
1–5 d	138/156 (88)
6–10 d	189/244 (77)
11–15 d	159/234 (68)
16–20 d	107/162 (66)
21–25 d	59/105 (56)

Note. PCR, polymerase chain reaction assay.

## Discussion

In this multicenter US study, we found that SARS-CoV-2 RNA shedding persists for >3 weeks in most patients with COVID-19. This finding has important implications for infection prevention in both inpatient and outpatient settings. The Centers for Disease Control and Prevention recommends 2 possible strategies for determining when isolation precautions can be discontinued for symptomatic patients with COVID-19: a symptom-based strategy and a test-based strategy.^4^ In the symptom-based strategy, isolation precautions can be discontinued 3 days after patient recovery and 10 days after symptom onset, whichever is longer. In the test-based strategy, isolation precautions can be discontinued after improvement in symptoms and at least 2 negative SARS-CoV-2 PCR tests collected at least 24 hours apart.^4^ Our findings that SARS-CoV-2 PCR tests remain positive for >3 weeks in most patients suggest that patients following the test-based strategy may remain on precautions for prolonged periods.

Our results are consistent with smaller studies that have also found prolonged duration of SARS-CoV-2 RNA positivity among patients with COVID-19.^1,2,5,6^ He et al^2^ examined the dynamics of viral shedding among 94 patients with COVID-19 and found that the SARS-CoV-2 tended to decrease below the detectable limit ~21 days after symptom onset. Xiao et al^5^ examined 56 patients with COVID-19 and found a median time from symptom onset to negative PCR test of 24 days. A positive PCR test does not necessarily correlate with viral transmissibility. Indeed, others have found no viable SARS-CoV-2 virus in culture among patients with prolonged SARS-CoV-2 RNA detection.^7–9^

Our study has several limitations. It was a retrospective cohort study among patients with COVID-19 who underwent SARS-CoV-2 PCR testing at the discretion of their medical providers. Patients with COVID-19 who are subsequently retested for SARS-CoV-2 are often inpatients being considered for transfer to a nursing home or other long-term care facility. These patients may be older and have more chronic medical conditions than COVID-19 patients in the outpatient setting, so our findings may not be representative of all individuals with COVID-19. We did not collect the clinical characteristics of patients in this analysis. In addition, we were not able to assess SARS-CoV-2 PCR test results in relation to the timing of symptom onset. Patients typically develop symptoms before they undergo their first SARS-CoV-2 PCR test. Therefore, our findings likely underestimate the duration of SARS-CoV-2 RNA shedding.

In conclusion, in a multisite cohort study, we found prolonged duration of SARS-CoV-2 RNA shedding among patients with COVID-19. More research is needed to understand the duration of SARS-CoV-2 transmissibility among patients with COVID-19.
